# An automatically contamination-avoiding technique for intracorporeal esophagojejunostomy using a transorally inserted anvil during laparoscopic total gastrectomy for gastric cancer

**DOI:** 10.1186/s12957-015-0563-0

**Published:** 2015-04-19

**Authors:** Yan-Feng Hu, Da Wang, Tian Lin, Ting-Yu Mou, Hao Liu, Tao Chen, Zhen-Wei Deng, Xin Lu, Jiang Yu, Guo-Xin Li

**Affiliations:** Department of General Surgery, Nanfang Hospital, Southern Medical University, No. 1838 The North Guangzhou Avenue, Guangzhou, 510515 Guangdong China; The Key Laboratory of Cancer Prevention and Intervention, China National Ministry of Education, Department of Surgical Oncology, The Second Affiliated Hospital of Zhejiang University School of Medicine, 88 Jie-Fang Road, Hangzhou, 310009 Zhejiang Province China

**Keywords:** Gastric cancer, Laparoscopy, Esophagojejunostomy, Gastrectomy

## Abstract

**Background:**

Intracorporeal Roux-en-Y esophagojejunostomy during laparoscopic total gastrectomy for gastric cancer remains a challenging manipulation due to the uncontrolled direction of the jejunal side or unintended embedded tissues, although several methods have been introduced. In this study, we simplified the procedure based on a surgical string fixing technique using a transorally inserted anvil (OrVil™; Covidien Ltd., Mansfield, MA, USA).

**Methods:**

From March 2012 to September 2013, 14 consecutive patients underwent simplified intracorporeal Roux-en-Y esophagojejunostomy using OrVil™ during laparoscopic total gastrectomy for gastric cancer at our hospital. Clinicopathologic characteristics and surgical outcomes of these patients were retrospectively analyzed.

**Results:**

All of the procedures were successful completed with no complication or conversion to open surgery. The mean overall operative time was 193.8 ± 41.8 min, whereas the mean reconstruction time was 32.6 ± 4.6 min. The mean estimated blood loss was 105.7 ± 65.4 ml. The mean diameter of anastomosis measured by upper gastrointestinal contrast X-ray test at 1 month after operation was 2.3 cm. During a median follow-up period of 12 months, neither local recurrence nor anastomosis-related morbidity was observed.

**Conclusions:**

Our preliminary results suggested that this automatically contamination-avoiding technique based on a surgical-string-fixing strategy using OrVil™ during laparoscopic total gastrectomy for gastric cancer might be feasible and safe and provide a simple solution for intracorporeal Roux-en-Y esophagojejunostomy.

## Background

Extracorporeal Roux-en-Y esophagojejunostomy during laparoscopy-assisted total gastrectomy for gastric cancer is one of the most challenging manipulations even in experienced hands. To overcome the surgical difficulty and maximize the clinical benefits from minimally invasive surgery in the management of patients with proximal gastric cancer, intracorporeal esophagojejunostomy, including either circular or linear anastomosis, has been introduced recently [1-4]. Among those methods, circular anastomosis following transabdominal insertion of an anvil with purse-string suture is the most frequently used pattern [1,4]. However, the difficulties in terms of laparoscopically creating a purse-string suture and fixing the anvil at the esophageal stump always exist, increasing the potential risk of complications and prolonging the operation time.

To avoid these difficulties mentioned above, a transorally inserted anvil (OrVil™; Covidien Ltd., Mansfield, MA, USA) device has been developed in recent years [5-7]. Previous reports [6,8] have shown its technical feasibility and potential advantages; however, the course of approximation between the anvil and center rod does not always proceed safely by intracorporeal laparoscopic view due to the uncontrolled direction of the jejunal side or unintended embedded tissues [7,8]. Thus, we attempted to simplify this technique by adopting several tips with inexpensive and available devices and evaluated its surgical safety in the present study.

## Methods

### Patients

Between March 2012 and September 2013, 14 consecutive patients with proximal gastric adenocarcinoma underwent simplified intracorporeal Roux-en-Y esophagojejunostomy using a transorally inserted anvil system (OrVil™; Covidien, Mansfield, MA, USA) during laparoscopic total gastrectomy at our hospital. These patients followed a stepwise postoperative management protocol for diet resume from water to other liquids to semi-fluids to normal food when the patient can tolerate the diet satisfactorily and is free from anastomotic complication as early as possible after surgery and received upper gastrointestinal contrast X-ray check of esophagojejunostomy at postoperative 1 month. Clinicopathologic characteristics and surgical outcomes of these patients based on a prospectively maintained database [9] were then retrospectively analyzed. The present study was approved by the Ethics Committee of Nanfang Hospital (No. 2013087A).

### Procedure before anastomosis

**Figure 1:**
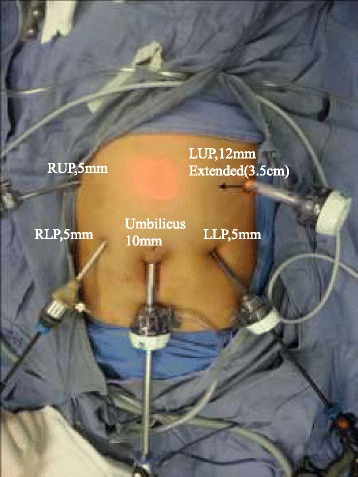
Placement of the trocars. The left upper port site will be extended transversely to an incision length of 3 to 4 cm for extraction of the specimen and insertion of the circular stapler before anastomosis. RUP, right upper port; LUP, left upper port; RLP, right lower port; LLP, left lower port.

Under general anesthesia, the patient was placed in the supine position with legs slightly apart (relaxed dorsal lithotomy position). The operator and assistant stood on the patient’s left and right side, respectively, and the camera holder stood between the legs of the patient. Trocar placement is shown in Figure 1.

**Figure 2:**
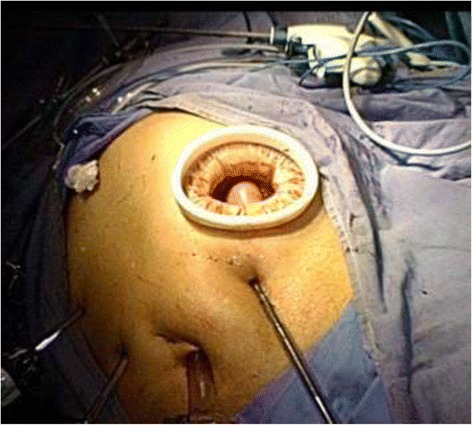
Minilaparotomy at the left upper quadrant for specimen retrieval and stapler insertion.

To obtain better exposure of the operative field, the falciform ligament and liver were suspended to the abdominal wall using Hyung’s strategy [4]. After dissection of the regional lymph nodes, the duodenal bulb was transected using a linear stapler laparoscopically, followed by transection of the distal esophagus with a proper distance from the lesion using a linear stapler with flexible articulation. Next, the entire specimen was removed through a 3- to 4-cm minilaparotomy incision extending to the left upper port site, where a wound protector (Beijing HangTian KaDi Technology R & D Institute, Beijing, China) was used (Figure 2).

### Anastomosis

#### Insertion of the anvil transorally

**Figure 3:**
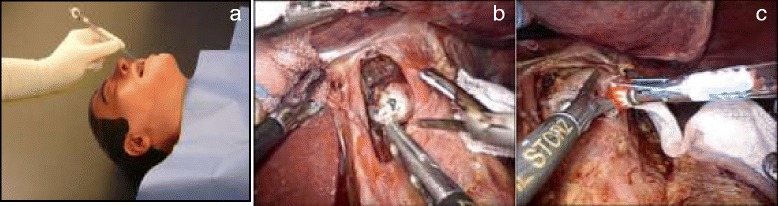
Placement of the anvil head. **(a)** The tube of the anvil head was inserted transorally (picture downloaded from the website of Covidien). **(b)** A small hole was made at the esophageal stump. **(c)** The thread was cut after fixation of the anvil head.

A small hole was created at one angle of the esophageal stump laparoscopically. Concurrently, an anesthetist inserted the tube of the anvil (OrVil™) transorally until the tip of the tube passed through the small hole at the esophageal stump (Figure 3a, b). A laparoscopic grasper was then used to slowly drag the tube until the anvil rod came into view.

The tube was lightly secured to fix the anvil at the proper position. The thread connecting the tube and anvil was then released (Figure 3c). The tube was removed to expose the rod of the anvil sufficiently. At that time, the insertion of the anvil was completed.

### Modification and tips of the intracorporeal esophagojejunostomy

**Figure 4:**
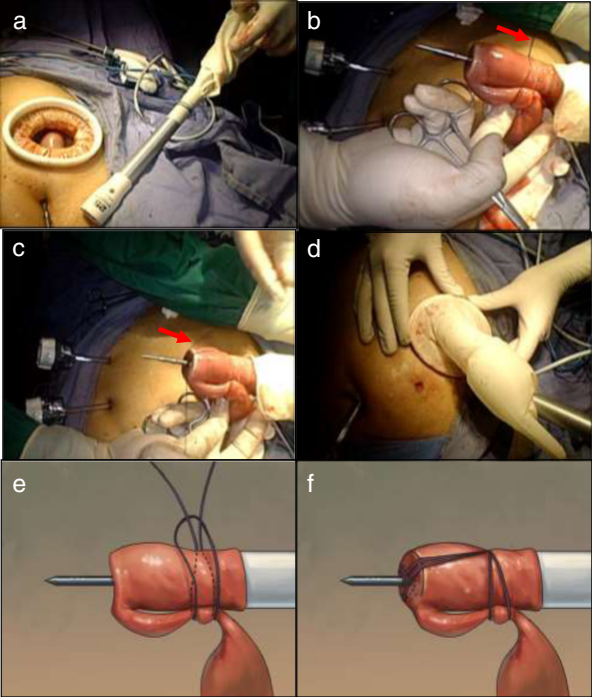
A self-made single-site access system. **(a)** The circular stapler passed through the glove. **(b)** The stapler, jejunal stump, and loop were fixed by the silk string. **(c)** A slipknot was made to fix the shaft. **(d)** Establishment of the pneumoperitoneum by the self-made single-site access system. **(e)** Schematic of making a slipknot using a silk string. **(f)** Schematic of making a surgical knot to the center rod. Red arrow points to the silk suture. The knot was released automatically during firing of the stapler without additional cutting.

The jejunum was transected 15 cm away from the Treitz’s ligament using a linear stapler laparoscopically. A surgical glove, a minilaparotomy wound protector, and the shaft of the stapler were integrated as a self-made single-site access system (Figure 4). The stapler was then positioned within the jejunal loop from the distal jejunal stump. Both the jejunal end and loop were anchored on the main unit with a silk string to create a slipknot to fix the center rod to prevent separation and avulsion (Figure 4).

### Approximation between the anvil and center rod

**Figure 5:**
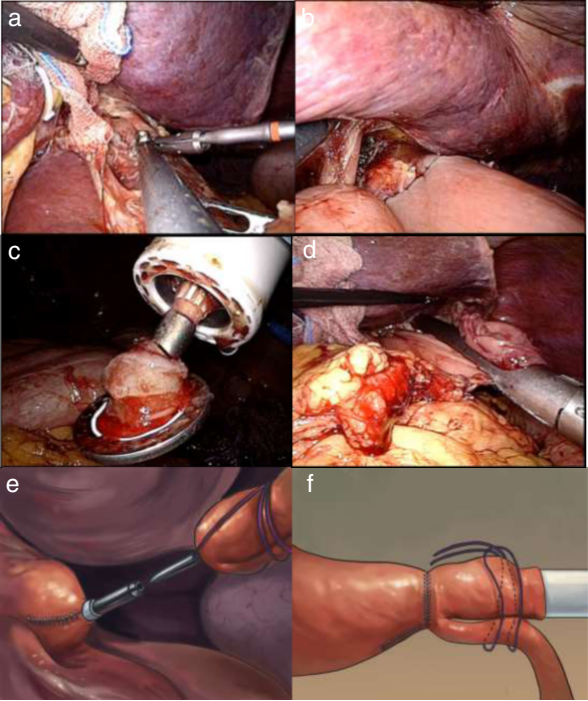
Intracorporeal anastomotic technique using a circular stapler. **(a)** Connection of the shaft and anvil. **(b)** Approximation of the shaft and anvil. The knot was released automatically during firing of the stapler. **(c)** The stapler was carefully removed. **(d)** The jejunal stump was closed using a linear stapler. **(e)** Schematic of approximation between the anvil and center rod. **(f)** Schematic of automatically removing an anchoring string during firing.

The anvil and circular stapler were connected, and anastomosis was performed under laparoscopic view directly (Figure 5). Moderation by rotating the stapler was easily performed to make a fine alignment, precisely controlling the direction of the jejunal side with little concern about tearing embedded tissues. After confirming that the bilateral crura of the diaphragm were not embedded in the stapler (the anastomotic stoma was satisfactory), the stapler was fired and loosened. Simultaneously, an anchoring string on the small bowel and the circular stapler were removed automatically without additional cutting. The circular stapler was removed gently from the anastomotic bowel, and the quality of the anastomosis was checked by identifying the donuts on the stapler. The jejunal stump was closed 2 to 3 cm away from the esophagojejunal site using a linear stapler. After side-to-side jejunojejunostomy was performed by linear stapler laparoscopically, the continuity of the digestive tract was completed.

### Statistical analysis

Demographic, operative, and clinicopathologic data of the patients are expressed as mean ± standard deviation or median (range) if they are continuous variables.

## Results

**Figure 6:**
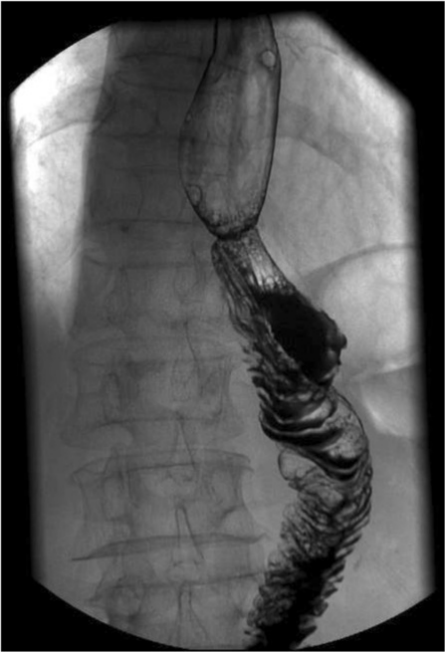
Upper gastrointestinal contrast X-ray check of esophagojejunostomy at postoperative 1 month.

The clinicopathologic features and operative results of the patients are shown in Table 1. The mean overall operation time was 193.8 ± 41.8 min. The mean reconstruction time was 32.6 ± 4.6 min. No intraoperative complication was observed. The mean first flatus time and hospital duration were 3.29 ± 0.73 and 8.7 ± 3.2 days, respectively. The mean diameter of anastomosis measured by upper gastrointestinal contrast X-ray test at 1 month after operation was 2.3 cm (Figure 6). During a median follow-up period of 12 months, neither local recurrence nor anastomosis-related morbidity was observed.

**Table 1:** **Clinicopathologic characteristics and operative results of the patients**

**Variable**	**Value**
Demography	
Male/female, *n*	10/4
Age, years	59.0 ± 12.1
Body mass index, kg/m^2^	23.4 ± 2.2
Operation	
Overall operation time, min	193.8 ± 41.8
Overall reconstruction time, min	32.6 ± 4.6
Time for anvil placement, min	8.4 ± 4.0
Time for esophagojejunostomy, min	23.8 ± 5.2
Estimated blood loss, ml	105.7 ± 65.4
Pathology	
Stage^a^ I/II/III, *n*	1/3/10
Proximal margin, cm	3.6 ± 1.7
No. of retrieved lymph nodes	33.9 ± 18.1
Immediate postoperative course	
First flatus, mean, days	3.3 ± 0.7
Hospital duration, mean, days	8.7 ± 3.2
Mortality, *n*	0
Short-term anastomosis-related complications, *n*	0
Esophagojejunostomy diameter, cm	2.3 ± 0.3
Follow-up	
Follow-up period (median, range), months	12 (6 to 24)
Long-term complications, *n*	0

Values are expressed as mean ± standard deviation unless otherwise indicated. ^a^According to the American Joint Committee on Cancer seventh edition.

## Discussion

Laparoscopic total gastrectomy becomes significantly challenging when the reconstruction procedure involves intracorporeal esophagojejunostomy because it is difficult to perform a proper purse-string suture at the esophageal stump and insert an anvil into the esophageal lumen [4]. Even more troublesome is applying the stapling device appropriately under limited laparoscopic views, particularly in obese patients [7]. Some reports have introduced solutions to improve the procedure, but problems remain such as the requirement for special expensive instruments that are not always available or additional skills [4,10,11]. A linear stapler has been chosen by some surgeons instead of a circular one to perform esophagojejunostomy laparoscopically that unsatisfactorily overcomes the limitation of requiring a certain esophageal length and an intracorporeal hand-suture [3].

To solve these problems and overcome the limitations, we describe herein a simplified technique for intracorporeal esophagojejunostomy using a circular stapler. No special technique is required, and the simplified procedure can be easily performed. Its safety and feasibility has been suggested in a series of 14 patients, of whom none suffered postoperative anastomosis-related complications.

Transoral insertion of an anvil (OrVil™) provides a solution for secure anvil placement in the esophagus. With an anesthesiologist’s help, pharyngeal or esophageal injury can be effectively avoided. Our method to manipulate the process is to open a small incision at the alternative edge of the esophageal stump after dividing the esophagus horizontally using a linear stapler and securing the anvil with a pair of grasping forceps after introducing the anvil tube into view. Thus, the anvil is guaranteed to be placed at an appropriate position without tearing the esophageal stump facilitating subsequent operations.

With similar methods in the recently published literature [7], problems such as distortion of the Roux limb or mesenterium and slipping of the esophagojejunal anastomotic site into the low mediastinum have not yet met ideal solutions. As presented above, fixation of the circular stapler and glove was followed by fixation of the jejunal loop and glove, causing the stapler-glove-jejunal loop to integrate and form a self-made single-site access system that prevents slippage of the jejunal loop out of the circular stapler during the intracorporeal procedure. Additionally, flexible mobilization is enabled to make a fine alignment, ensuring optimal functioning of the reconstructive digestive tract. The small bowel was more easily held backward to prevent it from becoming trapped between the anvil and circular stapler. The sealed space, established by turning over the glove edge to seal off the wound protector, allowed for a clearer visual field for the laparoscopic operation. Thus, tissue injury caused by excessive traction or slight errors can be minimized. Moreover, the anchoring suture could be cut along with the donut during the scalper firing and separated automatically without additional cutting when the main unit was pulled back. Thus, the simplified technique does not significantly create excessive steps and prolong the overall operative time. To prevent possible anastomotic stenosis, selection of the proper anvil size of circular stapler should be considered carefully, which usually depends on both the diameter of esophageal and jejunal stump. A diameter of 25-mm anvil is the most common choice at our institution for Chinese patients, while a relatively larger size would be applied casually. Meanwhile, intraoperative examination and postoperative early oral intake was also recommended [12]. In our daily clinical practice, generally, a stepwise management protocol for diet resume from water to other liquids to semi-fluids to normal food should be carried out when the patient can tolerate the diet satisfactorily and is free from anastomotic complication as early as possible after surgery. Theoretically, adding the solid food appropriately in terms of volume and frequency might be helpful to dilate the anastomotic site. Besides, routine upper gastrointestinal contrast X-ray test at postoperative 1 month is recommended for early detection of stricture.

## Conclusions

Our initial findings show that this automatically contamination-avoiding technique based on a surgical-string-fixing strategy is technically feasible and could provide a simple solution for intracorporeal Roux-en-Y esophagojejunostomy. However, a perspective study is needed to evaluate the surgical safety of this technique.
